# Nanostrucutured MnO_2_-TiN nanotube arrays for advanced supercapacitor electrode material

**DOI:** 10.1038/s41598-022-05167-1

**Published:** 2022-02-08

**Authors:** Peng Ren, Chao Chen, Xiuchun Yang

**Affiliations:** 1 Shanghai Key Laboratory of R&D for Metallic Functional Materials, Tongji University, Shanghai, 201804 People’s Republic of China; 2 School of Chemistry and Civil Engineering, Shaoguan University, Shaoguan, 512005 People’s Republic of China; 3 Key Laboratory of Advanced Civil Engineering Materials of Ministry of Education, Tongji University, Shanghai, 201804 People’s Republic of China; 4 School of Materials Science and Engineering, Tongji University, Shanghai, 201804 People’s Republic of China

**Keywords:** Supercapacitors, Nanoscale materials

## Abstract

The capacitance of MnO_2_ supercapacitors (SCs) is not high as expected due to its low conductivity of MnO_2_. The synergistic effects of MnO_2_ with high theoretical specific capacitance and TiN with high theoretical conductivity can extremely enhance the electrochemical performance of the MnO_2_-TiN electrode material. In this work, we synthesized different nanostructured and crystalline-structured MnO_2_ modified TiN nanotube arrays electrode materials by hydrothermal method and explained the formation mechanism of different nanostructured and crystalline-structured MnO_2._ The influences of MnO_2_ nanostructures and crystalline-structures on the electrochemical performance has been contrasted and discussed. The specific capacitance of δ-MnO_2_ nanosheets-TiN nanotube arrays can reach 689.88 F g^−1^, the highest value among these samples TN-MO-SS, TN-MO-S, TN-MO-SR, TN-MO-RS, and TN-MO-R. The reason is explained based on MnO_2_ nanostructure and crystalline-structure and electron/ion transport properties. The specific capacitance retention rates are 97.2% and 82.4% of initial capacitance after 100 and 500 cycles, respectively, indicating an excellent charging-discharging cycle stability.

## Introduction

With the development of renewable energy, high-performance electrochemical energy storage will take into account in the future. A device with an energy storage function called "Laihton bottle" was discovered by Dutch in 1746^1^. Since then, the mystery of capacitors has been gradually revealed. The research on supercapacitors(SCs) can be traced back to 1879 when Helmholz first discovered the characteristics of electric double-layer capacitance at the electrochemical interface^2^. In 1957, Becker applied for the first carbon electrode supercapacitor patent, which has a similar energy density to batteries and has a specific capacitance that is 3 to 4 orders of magnitude higher than ordinary capacitors^3^. In the researches on SCs, the electrode materials have an important effect on performance.

Generally, SCs can be divided into electrical double-layer capacitors (EDLCs) and pseudocapacitors (PCs) depending on their energy storage mechanism^4^, EDLCs mainly based on high surface area materials, such as carbon, graphene^5^, graphite oxide^6^ so on, which are all kinds of nanostructures, PCs mainly based on metal oxides and graphene-like layered metal compounds^7^, using transition metal oxide (Co_3_O_4_, NiO, RuO_2_ and MnO_2_, etc.) nanomaterials with good electrochemical properties is a practical way to optimize the electrochemical performance of the electrode materials for the development of high-performance SCs^8^.

MnO_2_ is considered as a potential electrode material for SCs, which not only possesses electric double layer capacitance but also has high pseudocapacitance capacity as a semiconductor. The theoretical specific capacitance of MnO_2_ can reach 1370 F g^−1^^9,10^. In 1999, Lee and Goodenough first researched the pseudocapacitance properties of MnO_2_ in an aqueous solution and proposed that the main energy storage mechanism is the pseudocapacitance reaction in the electrode material^11^. The charge/discharge processes mainly include the adsorption/desorption of metal cations on the surface of MnO_2_ and the intercalation/de-intercalation in MnO_2_ with rapid and reversible redox reactions^12,13^. In addition, the crystal structure of MnO_2_ directly impacts electrochemical performance. Brousse et al. prepared MnO_2_ with different crystal structures and studied their electrochemical properties^14^. The results showed that the specific capacitances of one-dimensional α-MnO_2_ and two-dimensional δ-MnO_2_ are about 110 F g^−1^, respectively. Ghodbane et al. further studied MnO_2_ electrode materials with different crystal structures and proposed that the specific capacitances of the λ-MnO_2_ and δ-MnO_2_ with three-dimensional structures are 241 F g^−1^ and 225 F g^−1^, respectively^15^. The capacitance of the SCs with simple MnO_2_ electrode is not high as expected due to the low conductivity of MnO_2_ (10^−3^ ~ 10^−4^ S m^−1^)^16^. Therefore, MnO_2_ needs to be compounded with other materials with good electrical conductivity to improve the overall electrochemical performance including specific capacitance, charge/discharge performance, and cycle characteristics, researchers have made many attempts to prepare supercapacitor electrodes by mixing MnO_2_ with highly conductive materials^17–24^, Since transition metal nitrides have great electrical conductivity, electrochemical characteristics, chemical stability and long service life, TiN, VN, WN, CrN and TiVN are widely used as electrode materials of SCs^25–27^. TiN has been used in electric devices such as microelectronics, semiconductor device electrodes, lithium ion batteries, fuel cells and SCs as a low-cost transition metal nitride with good conductivity (4000 ~ 55,500 s cm^−1^) and stability^28–30^. Tang et al. used urea and TiCl_4_ to prepare TiN as a SCs electrode material with a specific capacitance of 407 F g^−1^^31^.

In this work, MnO_2_ nanosheet spheres, nanosheets, nanorod spheres, and nanorods are synthesized on TiN nanotube arrays for obtaining an electrode material for SCs, where the nanostructured MnO_2_ is more chemically stable than MoS_2_^32^ and has better electrochemical performance than layered MnO_2_^33^. The composition and morphology are measured by using XRD, SEM and EDS. The electrochemical performances of all samples in an electrolyte containing K^+^ are measured and discussed.

## Methods

### Preparation of TiO_2_ NTAs on mesh (TONM)

All reagents are analytical grade and used without further purification. A large piece of raw Ti mesh (50 meshes, 99.5% purity) with a thickness of 0.12 mm was cut into square pieces of 2.5 × 2.5 cm^2^, which were ultrasonically degreased in acetone, isopropanol, and methanol for 15 min, respectively, then chemically etched in a mixture of HF and HNO_3_ aqueous solution (HF:HNO_3_:H_2_O = 1:4:10 in volume, total 20 mL) for 10 s, afterward rinsed with deionized water and finally dried in air. Electrochemical anodic oxidation was performed at 60 V direct current voltages for 24 h in DEG solution containing 1.5 vol.% HF, using Ti mesh as the working electrode and Pt plate as a counter electrode. The as-prepared samples were ultrasonically rinsed with deionized water and dried in the air^33^.

### Preparation of TiN NTAs on mesh (TNNM)

TONM samples in a quartz boat were placed in the heating center of a horizontal quartz tube vacuum furnace. Prior to heating, the system was evacuated and flushed with high pure N_2_ to eliminate oxygen. Afterward, the furnace was heated in N_2_ to 750 °C, and then changed to NH_3_ flow keeping a flow rate of 100 mL/min for 5 h while the temperature was maintained. Finally, the furnace cooled down to room temperature in N_2_.

### Preparation of MnO_2_ modified TNNM

Different precursor solutions were employed for synthesizing MnO_2_ nanostructures by hydrothermal synthesis method. A TNNM sample was placed at the bottom of the reaction solution in a sealed 150 mL Teflon-lined autoclave, which was put into a muffle furnace for hydrothermal reaction. The solution compositions and reaction solutions are summarized in Table 1.

**Table 1:** The precursor solution compositions and conditions of hydrothermal reaction for preparing nanostructured MnO_2_.

Solution	KMnO_4_	MnSO_4_·H_2_O	HCl	H_2_O	Temperature [°C]	Time [h]	Sample
M-1	0.875 g	0.35 g	–	70 mL	140	3	TN-MO-SS
						12	TN-MO-S
						18	TN-MO-SR
M-2	1.106 g	–	0.88 mL	70 mL	150	6	TN-MO-RS
M-3	0.7875 g	–	1.75 mL	70 mL	150	12	TN-MO-R

### Characterization

The crystalline phase compositions of the samples were measured by a Rigaku D/Max 2550VB3 + /PC X-ray diffractometer (XRD) equipped with graphite monochromatized Cu Kα radiation (λ = 0.15405 nm). Nanostructures and elemental distributions of the samples were characterized by a Schottky field emission scanning electron microscopy (FESEM, FEI Nova NanoSEM 450) equipped with energy dispersive spectroscope (EDS, EDAX).

### Electrochemical performance measurement

Electrochemical measurement was measured by CHI 660E electrochemical system using a three-electrode system where the samples as a working electrode, Pt foil as a counter electrode, and Ag/AgCl electrode as a reference electrode in 2 mol/L KCl solution. Cyclic voltammetry (CV) curves were obtained in a voltage range from − 0.2 V to 0.8 V at different scan rates of 5, 10, 20, 40, 60, 80 and 100 mV s^−1^, respectively. Galvanostatic charge/discharge curves were recorded in a potential window from − 0.2 V to 0.8 V at a series of current densities. The electrochemical impedance spectroscopy (EIS) was conducted in the frequency from 100 kHz to 10 mHz at an open-circuit potential vibration of 5 mV^33^.

## Results and discussion

**Figure 1:**
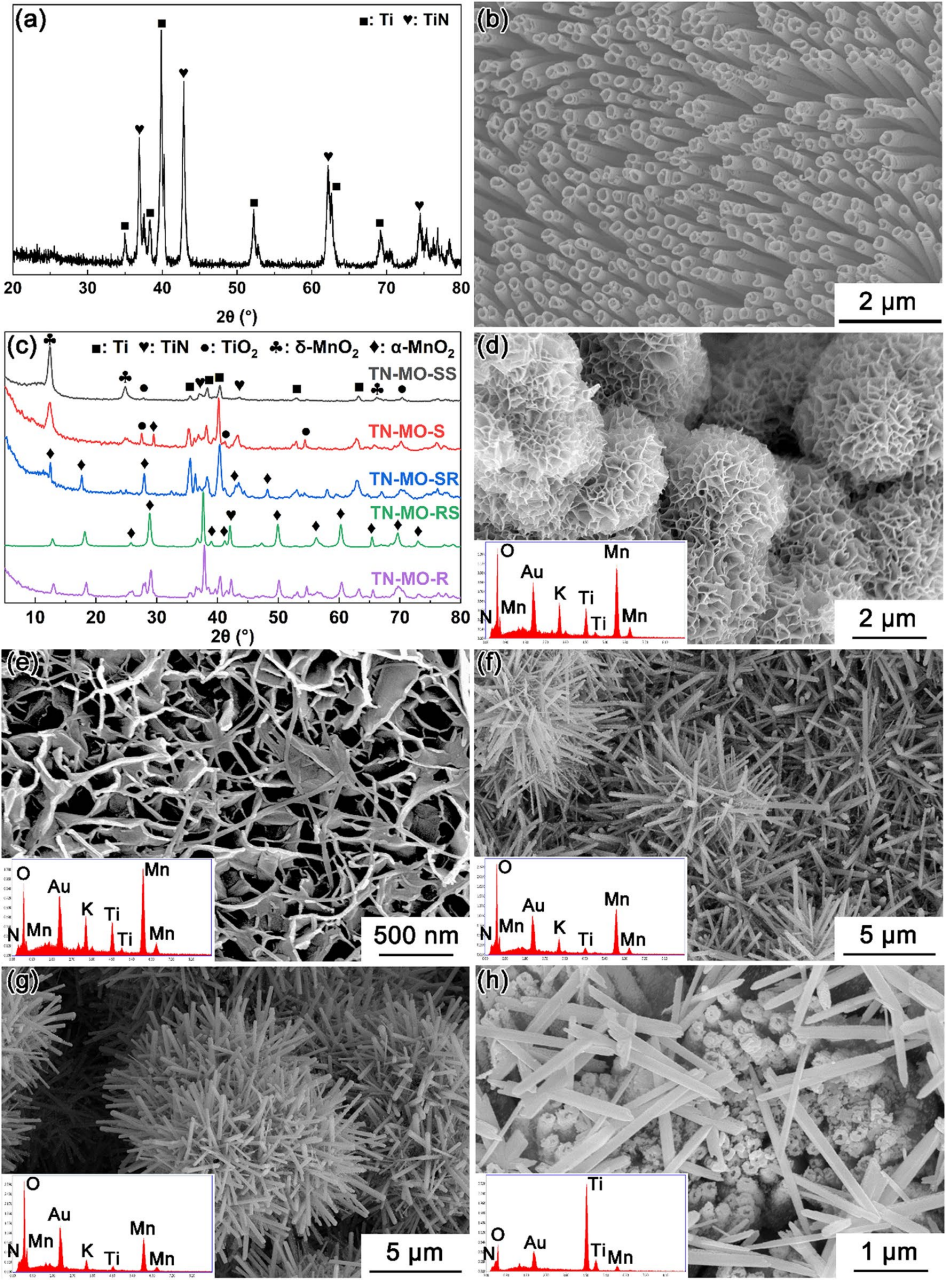
XRD pattern (**a**) and SEM image (**b**) of TNNM. XRD patterns (**c**) and SEM images (**d**–**h**, the insets are EDS spectra) of TN-MO-SS, TN-MO-S, TN-MO-SR, TN-MO-RS, and TN-MO-R.

**Figure 2:**
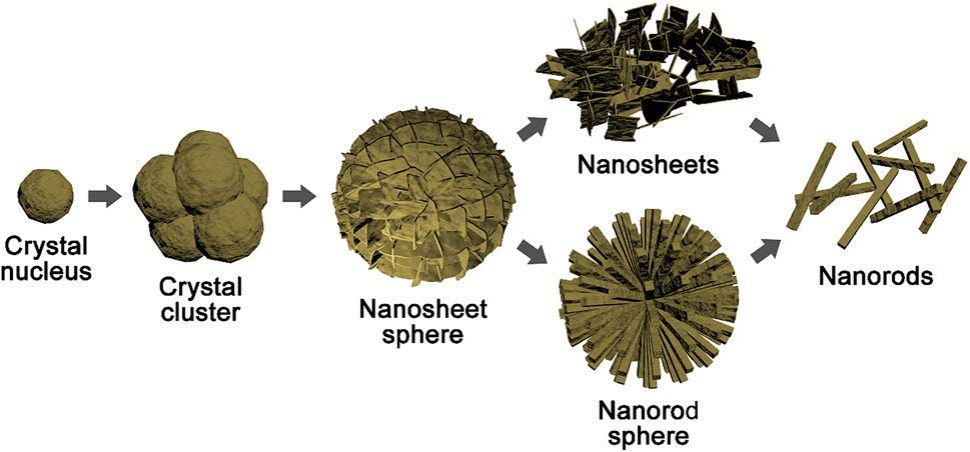
Schematic diagrams of MnO_2_ nanostructure growth process.

Figure 1a indicates that TNTM consists of Ti (JCPDS card No. 65-3362) and TiN (JCPDS card No. 65-5759). Ti is from Ti mesh and TiN is from the high-temperature ammonolysis of anodic TiO_2_. Figure 1b indicates that the TiN is vertically aligned TiN nanotubes. As shown in the XRD patterns of TN-MO-SS, TN-MO-S, TN-MO-SR, TN-MO-RS, and TN-MO-R (Fig. 1c), TN-MO-SS and TN-MO-S mainly contain δ-MnO_2_ crystals (JCPDS card No. 80-1098), while the MnO_2_ in TN-MO-SR, TN-MO-RS, and TN-MO-R is α-MnO_2_ (JCPDS card No. 44-0141). In addition, TN-MO-S also contains a little α-MnO_2_. According to the SEM images in Fig. 1d–h, the MnO_2_ nanostructures in TN-MO-SS, TN-MO-S, TN-MO-SR, TN-MO-RS and TN-MO-R are nanosheet spheres, nanosheets, nanorods, nanorod spheres and dispersed nanorods, respectively. The EDS spectra in Fig. 1d–h insets further demonstrate the compositions of all samples. Equations (1) and (2) depict the chemical reactions for generating MnO_2_ in M-1 (Eq. (1)), M-2 and M-3 (Eq. (2)) solutions^34,35^. Figure 2 shows the crystal growth process under hydrothermal reaction conditions. At first, a number of crystal nuclei rapidly form in the solution, which aggregates into nanoparticles. Afterward, nanosheets grow through the Ostwald ripening mechanism around the nanoparticles due to the particular lamellar crystal structure of δ-MnO_2_ and the intercalation of K^+^. As the hydrothermal reaction continues, the nanosheet spheres gradually disintegrate and form the intercalated nanosheets. Meanwhile, since α-MnO_2_ is more stable than δ-MnO_2_ thermodynamically, the δ-MnO_2_ phase begins to transform into the α-MnO_2_ phase with the α-MnO_2_ nuclei generating in the δ-MnO_2_ nanosheets. Then, the δ-MnO_2_ crystal domains diffuse to the α-MnO_2_ nucleus and convert into α-MnO_2_, while α-MnO_2_ nanorods grow through the Ostwald ripening mechanism^34,36^. In M-2 and M-3 solutions, the strong reducibility of Cl^**−**^ and the presence of H^+^ greatly accelerate the chemical reaction and phase transition speed^34^.

1 $${3Mn}^{2+}+{2MnO}_{4}^{-}+{2H}_{2}O\to {5MnO}_{2}+{4H}^{+}$$

2 $${2MnO}_{4}^{-}+{8H}^{+}+{6Cl}^{-}\to {2MnO}_{2}+{3Cl}_{2}+{4H}_{2}O$$

**Figure 3:**
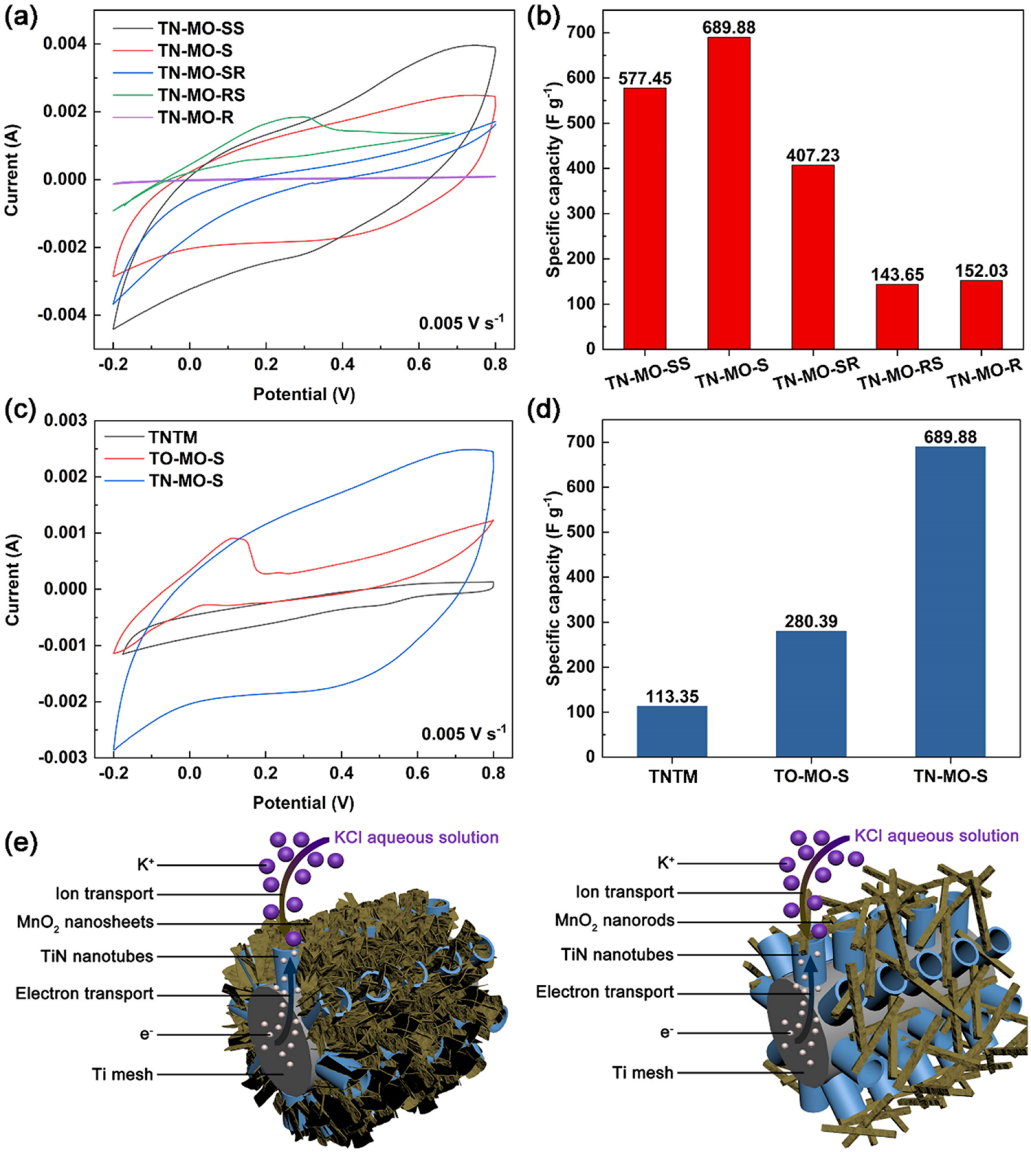
Cyclic voltammetry curves (**a**) and corresponding specific capacitances (**b**) of TN-MO-SS, TN-MO-S, TN-MO-SR, TN-MO-RS, and TN-MO-R with the sweep speed of 5 mV s^−1^. Cyclic voltammetry curves (**c**) and corresponding specific capacitances (**d**) of TNNM, TO-MO-S and TN-MO-S with the sweep speed of 5 mV s^−1^. Schematic diagrams of nanostructures of TN-MO-S and TN-MO-R (**e**).

**Figure 4:**
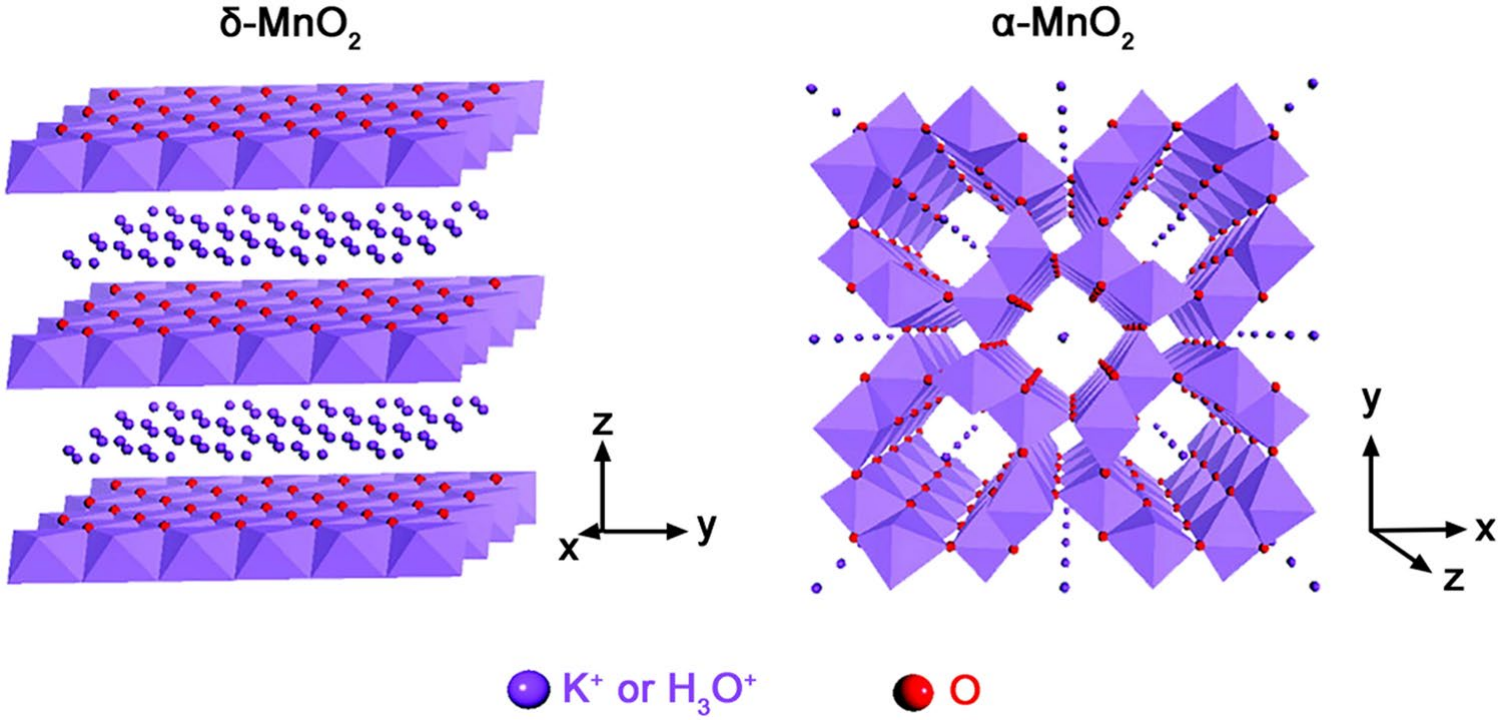
Schematic diagrams of δ-MnO_2_ and α-MnO_2_ crystal structure.

Figure 3a,b show the cyclic voltammetry curves with the sweep speed of 5 mV s^−1^ and corresponding specific capacitances of samples TN-MO-SS, TN-MO-S, TN-MO-SR, TN-MO-RS, and TN-MO-R. TN-MO-S has the largest specific capacitance of 689.88 F g^−1^. The specific capacitances of TN-MO-SS, TN-MO-SR, TN-MO-RS and TN-MO-R are 577.45 F g^−1^, 407.23 F g^−1^, 143.65 F g^−1^ and 152.03 F g^−1^, respectively. Figure 3c,d show the cyclic voltammetry curves with the sweep speed of 5 mV s^−1^ and corresponding specific capacitances of TNTM, TO-MO-S and TN-MO-S. Obviously, the specific capacitance of TN-MO-S is about 6.1 times and 2.5 times of TNTM and TO-MO-S, respectively. The results demonstrate that the synergistic effects of MnO_2_ nanosheets and TiN nanotube arrays significantly increase the specific capacitance. The specific capacitance mainly depends on the surface area of MnO_2_ and the capacity of K^+^^37^. TN-MO-S and TN-MO-SS have large specific surface area and great capacity for K^+^ due to layered crystal structure (Fig. 4)^11^. The 3D structures formed by the intercalation of nanosheets benefit energy storage with electrolyte ions intercalation/de-intercalation and provide numerous chemical reaction sites. In addition, the contact between MnO_2_ nanosheets and TiN nanotubes is more sufficient and tighter than that of MnO_2_ nanorods, which facilitates the transport of electrons between the substrate and the active substance (Fig. 3e). Since the hydrothermal reaction time during the preparation of TN-MO-S is longer than TN-MO-SS, TN-MO-S contains more hydrates to adsorb more K^+^ than TN-MO-SS, which further improves the pseudo-capacitance. TiN nanotube arrays can not only provide high-speed channels for electron transport, but also expands the specific surface area as a support for active substances providing more space for the ion intercalation/de-intercalation during the electrochemical process. Besides, TiN nanotube arrays directly contact with the substrate without the requirement of adhesion agent, which efficiently promotes the charge transfer between the interface.

**Figure 5:**
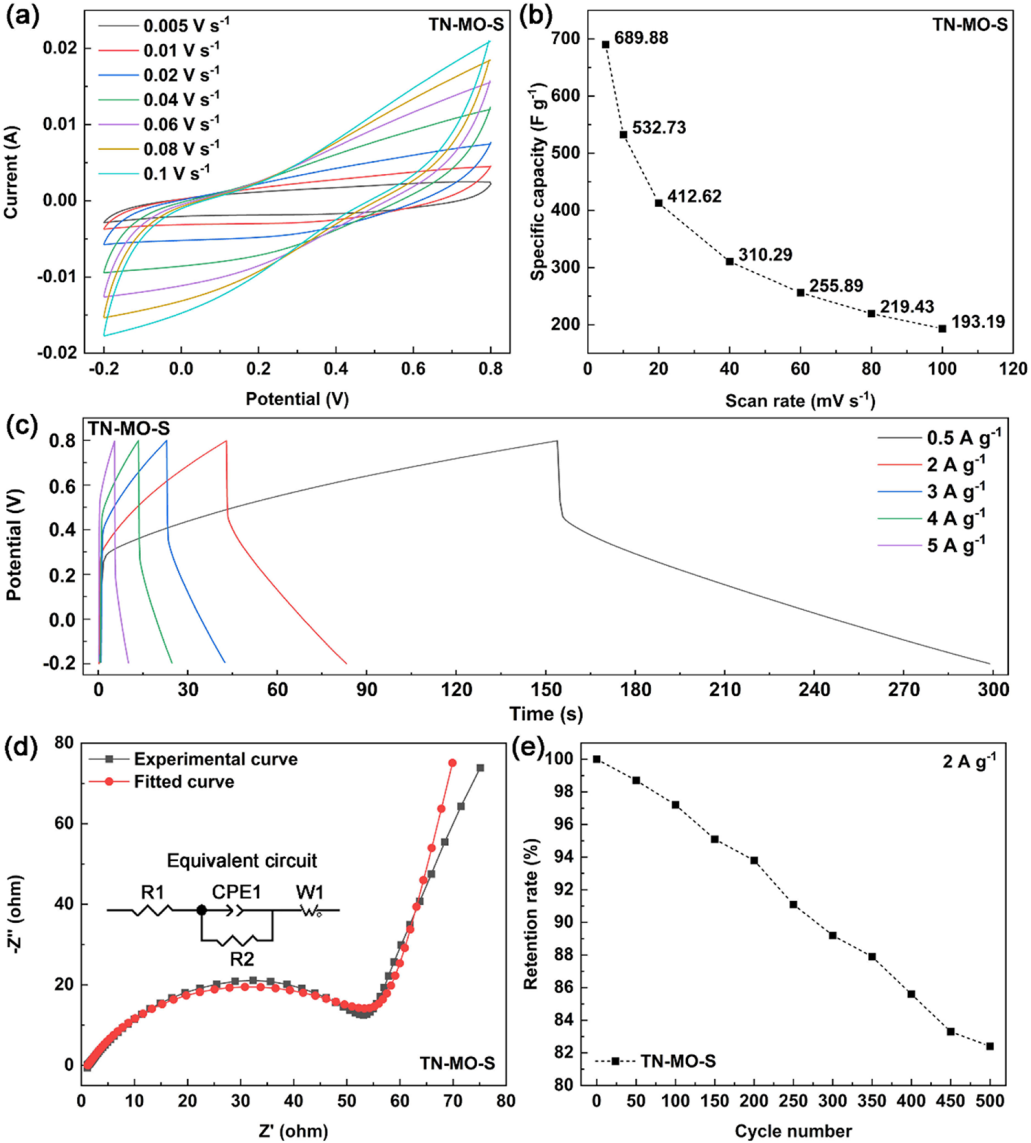
Cyclic voltammetry curves with different scan rates (**a**), corresponding specific capacitances (**b**), charge–discharge curves with different current densites (**c**), Nyquist plot and fitted curve (**d**, the inset shows the equivalent circuit), and the cyclic stability (**e**) of TN-MO-S.

**Figure 6:**
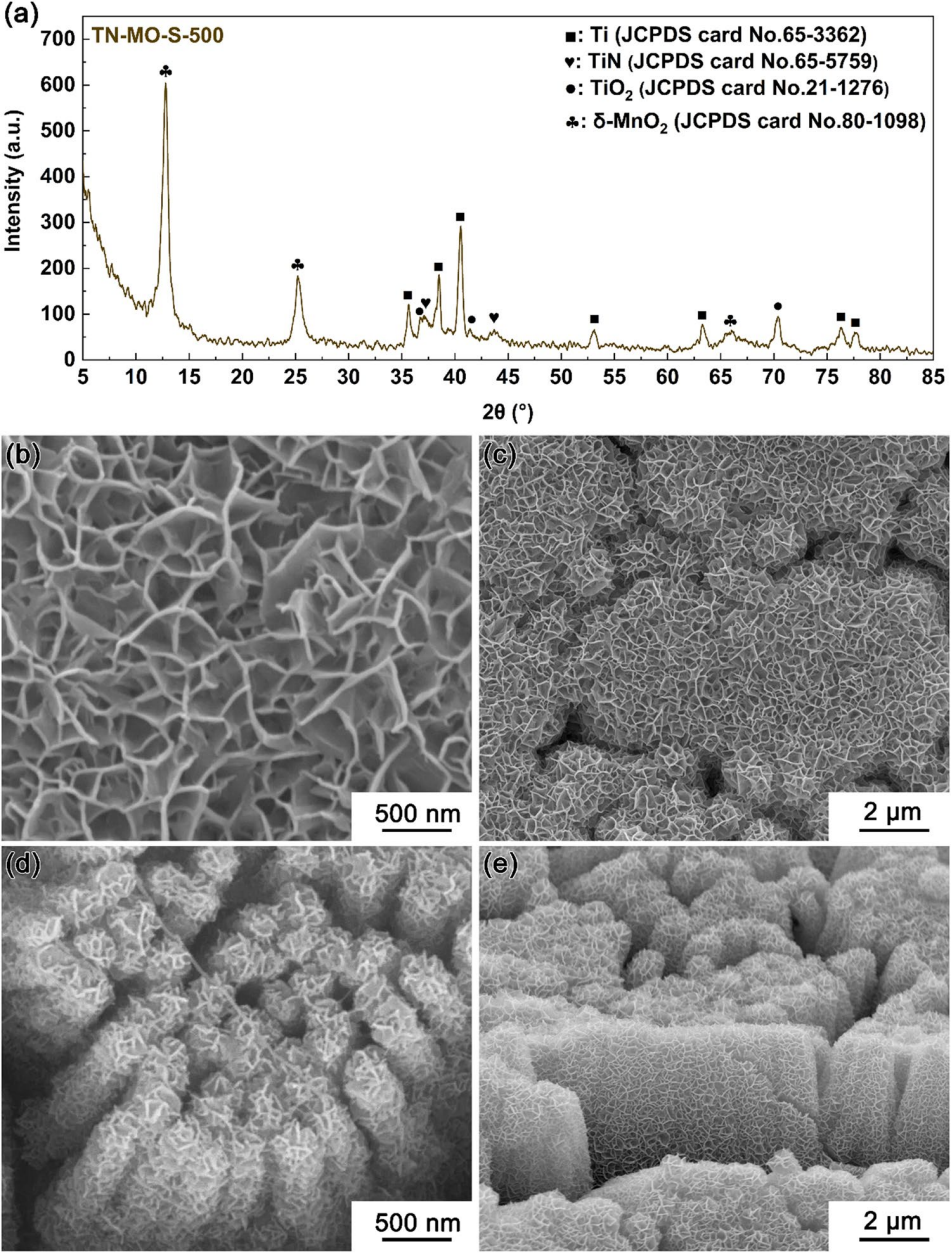
XRD pattern (**a**) and SEM images (**b**–**e**) of TN-MO-S-500.

Figure 5a,b show the curves and corresponding specific capacitance of TN-MO-S at different scan rates. The cyclic voltammetry curves maintain symmetrical shapes from 0.005 V s^−1^ to 0.1 V s^−1^, indicating the magnification capacity of the electrode material. The specific capacitance decreases with the increase of scan rate because of the insufficient Faraday reaction time at a high scanning rate. Figure 5c shows the charging-discharging curves of TN-MO-S at different current densities. The nearly symmetrical triangular outlines manifest the capacitive and reversible characters of the electrode. The Nyquist plot, corresponding fitted curve and the equivalent circuit of TN-MO-S is shown in Fig. 5d. The internal resistance (R1) and the charge transfer resistance (R2) of the electrode are low as 1.183 Ω and 52.23 Ω, respectively, indicating excellent electronic conductivity and electron diffusion. Figure 5e depicts the cycle stability of TN-MO-S by charging-discharging measurements at a current density of 2 A g^−1^ for consecutive 500 cycles. The specific capacitance of the electrode maintains 97.2% and 82.4% of initial capacitance after 100 and 500 cycles, respectively. Figure 6 shows the composition and morphology of TN-MO-S after 500 charging-discharging measurement cycles. Generally, the composition and morphology hardly change as shown in Fig. 6a–c. Meanwhile, as shown in Fig. 6d,e, the amount of MnO_2_ nanosheets deposited in some areas of the sample is reduced, indicating that the loss of the active substance is the main reason for the specific capacitance attenuation. However, it can be observed in Fig. 6d,e that MnO_2_ nanosheets firmly and uniformly grow on not only the nanotube array surface but also the walls of nanotubes. The close integration of MnO_2_ nanosheets and TiN nanotubes improves the transportation of electrons and ions so that TN-MO-S has great electrochemical performance as a SCs electrode.

## Conclusions

In summary, various MnO_2_ nanostructures are synthesized on TiN nanotube arrays by hydrothermal method including nanosheet spheres, nanosheets, nanorod spheres, and nanorods for developing an advanced electrode material of SCs. The TiN nanotubes with excellent conductivity and great specific surface area provide highly efficient paths for charge transport and more electrochemical reaction sites. The nanostructured MnO_2_ with high theoretical specific capacitance of 1370 F g^−1^ improves the pseudocapacitance reaction and specific capacitance of the electrode material. The specific capacitance of δ-MnO_2_ nanosheets-TiN nanotube arrays can reach 689.88 F g^−1^ because of its good magnification capacity and its excellent electronic conductivity and electron/ion transport properties. Its specific capacitance retention rate is 97.2% and 82.4% of initial capacitance after 100 and 500 cycles, respectively, indicating a good charging-discharging cycle stability. Hence, the synergistic effect of TiN and MnO_2_ can extremely enhance the electrochemical performance of the electrode material for SCs.

## Data Availability

All data included in this study are available upon request by contact with the corresponding author.
